# A Case Report of Surgical and Postoperative Treatment for Thyroid Angiosarcoma with Pulmonary Metastasis

**DOI:** 10.70352/scrj.cr.25-0349

**Published:** 2025-11-15

**Authors:** Gai Inaguma, Takahiro Ichikawa, Dai Takeuchi, Yuko Takano, Madoka Iwase, Reiko Ohata, Kayoko Sugino, Mariko Asai, Yumiko Akita, Misato Yamamoto, Yuri Ozaki, Nao Torii, Chihiro Toyoda, Misaki Hatasa, Norikazu Masuda, Toyone Kikumori

**Affiliations:** 1 Department of Breast and Endocrine Surgery, Nagoya University Hospital, Nagoya, Aichi, Japan; 2 Department of Breast Oncology, Aichi Cancer Center, Nagoya, Aichi, Japan; 3 Department of Breast Surgery, Toyohashi Municipal Hospital, Toyohashi, Aichi, Japan; 4 Department of Breast Surgery, Graduate School of Medicine, Kyoto University, Kyoto, Kyoto, Japan

**Keywords:** thyroid angiosarcoma, pulmonary metastasis, paclitaxel, thyroidectomy, comprehensive genome profiling

## Abstract

**INTRODUCTION:**

Thyroid angiosarcoma (TAS) is an extremely rare and highly aggressive cancer, representing less than 1% of all sarcomas. Typically diagnosed in individuals aged 50–80, with a higher incidence in women, TAS has a poor prognosis due to its tendency to metastasize, leading to a low 5-year survival rate. Due to its rarity, standardized treatment approaches are lacking, often involving a combination of surgery, chemotherapy, and radiation. This report presents TAS in a Japanese male.

**CASE PRESENTATION:**

A 78-year-old male with pre-existing conditions presented with a 30-year history of a slow-growing thyroid tumor that exhibited rapid enlargement 4 months prior to surgery. Initial fine-needle aspiration cytology was no malignancy. Examinations revealed a firm, poorly mobile 10 cm mass in the anterior neck, and left pleural effusion. Notably, papules developed at the aspiration site and progressively enlarged. Preoperative imaging indicated a malignant thyroid tumor, prompting a right thyroid lobectomy with resection of anterior neck muscles and overlying skin. Histological analysis confirmed a highly hemorrhagic angiosarcoma with infiltration into surrounding tissues. Immunohistochemical findings supported the diagnosis of TAS. A comprehensive genomic profiling testing yielded no specific therapeutic recommendations. Paclitaxel therapy was initiated 2 months after surgery, resulting in the shrinking of pulmonary nodules and the decreasing left pleural effusion. The patient subsequently died from septic shock due to a urinary tract infection 5 months after starting chemotherapy, without evidence of cervical recurrence or neutropenia during treatment.

**CONCLUSIONS:**

This case highlights the perioperative management of a rare primary TAS. In the absence of established treatment guidelines, surgical resection followed by paclitaxel administration could be a potential therapeutic strategy to control disease progression. An accumulation of case reports is needed to better understand this aggressive malignancy and to facilitate the development of optimized therapeutic strategies.

## Abbreviation

EMA
: epithelial membrane antigen
FDG
: ^18^F-fluorodeoxyglucose
HE
: hematoxylin-eosin
PAX8
: paired box 8
TAS
: thyroid angiosarcoma
TTF-1
: thyroid transcription factor-1
UTI
: urinary tract infection

## INTRODUCTION

TAS represents an exceedingly rare and highly aggressive malignancy, accounting for less than 1% of all sarcomas.^1,2)^ Regional clustering has been observed in the Alpine region, suggesting associations with iodine deficiency and multinodular goiter. External factors, including exposure to radiation and vinyl chloride, have also been implicated as potential etiologic agents.^3–5)^ The typical age of onset ranges from 50 to 80 years, with a female predominance. The prognosis for TAS is generally poor, characterized by a propensity for metastasis to the cervical lymph nodes, lungs, and bone marrow, resulting in a reported 5-year survival rate of 33.3%.^6,7)^ Treatment strategies for TAS are not standardized, often involving a multimodal approach combining surgery, chemotherapy, and radiation therapy. Herein, we report a case of perioperative management of primary TAS in a Japanese male patient.

## CASE PRESENTATION

**Fig. 1:**
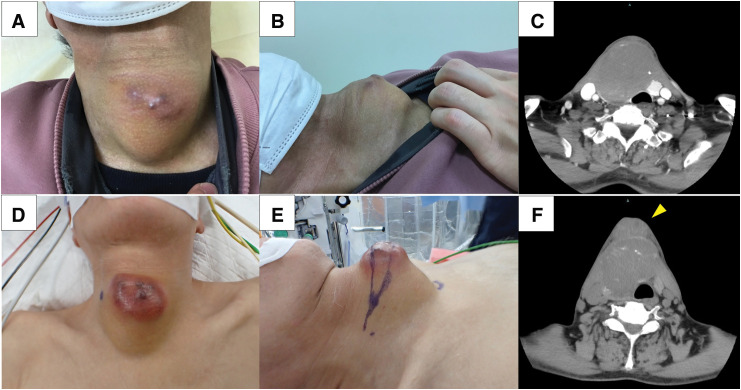
(**A**, **B**) A mass with some papules was on the neck at 1st visit. (**C**) A round mass abutted the left thyroid lobe and showed no obvious invasion to adjacent tissue at 1st visit. (**D**, **E**) The papules developed by aspiration cytology were enlarged and the surrounding redness had spread over 2 months after 1st visit. (**F**) The space between the tumor and the anterior neck skin has thickened (yellow arrowhead).

**Fig. 2:**
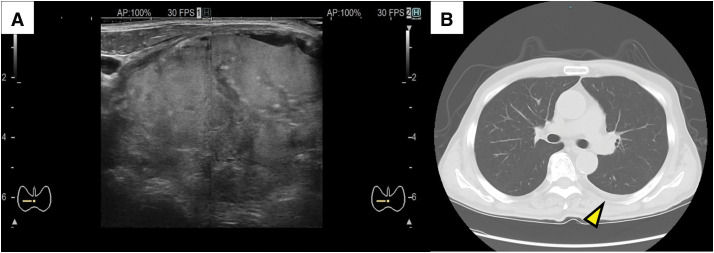
(**A**) Ultrasonography at the anterior neck showed a heterogeneous mass. (**B**) CT showed left pleural effusion (yellow arrowhead), but no pulmonary nodules.

A 78-year-old male with a medical history significant for diabetes mellitus, depression, cataracts, glaucoma, and benign prostatic hyperplasia. His family history was remarkable for Graves’ disease in his sister and colon cancer in his brother. There was no familial history of other endocrine disorders or malignancies. He had a thyroid tumor for approximately 30 years, which was soft, slowly growing, and asymptomatic. But no cytology and image tests had been performed for the tumor. Four months before surgery, the thyroid tumor became firm and rapidly enlarged, and he visited a local hospital. Fine-needle aspiration cytology was performed but did not detect malignant cells. Two months before surgery, he was referred to our institution. On the initial examination, a hard, poorly mobile tumor approximately 10 cm in diameter was in his anterior neck (**Fig. 1A** and **1C**). Papules were present on the skin of his anterior neck at the site of fine-needle aspiration (**Fig. 1B**). The papules had increased in size by the day of surgery (**Fig. 1D**–**1F**). No obvious hoarseness was noted. Thyroid function tests were within normal limits (**Table 1**). The cervical ultrasonography revealed a well-defined, mixed mass in his right neck (**Fig. 2A**). No obvious cervical lymphadenopathy was observed. CT showed a tumor in the right thyroid lobe and a small amount of left pleural effusion (**Fig. 2B**), but no apparent lymph node or distant metastases. The preoperative diagnosis was a malignant tumor, such as anaplastic thyroid carcinoma. Due to a clear boundary between the tumor, the left thyroid lobe, and surrounding tissues, and the significant compressive symptoms caused by the tumor enlargement, a right thyroid lobectomy was planned.

**Table 1:** Thyroid function before surgery

Parameter	Value	SI Unit
TSH	0.2847	µIU/mL
Free T3	2.64	pg/mL
Free T4	1.1	ng/mL
Thyroglobulin	36.1	ng/mL
Anti-thyroglobulin antibody	16.2	IU/mL
PTH-intact	68.3	pg/mL

Free T3, free triiodothyronine; Free T4, free thyroxine; PTH-intact, parathyroid hormone-intact; TSH, thyroid stimulating hormone

**Fig. 3:**
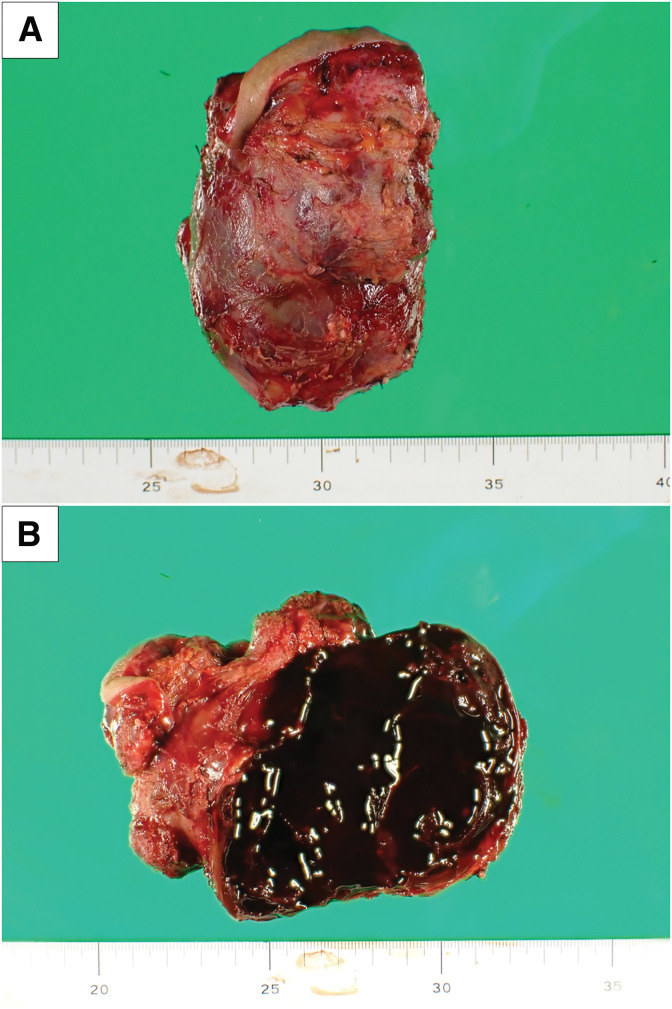
(**A**) A 10-cm tumor with neck skin was removed, and it had a clear boundary and no capsule damage. (**B**)The internal cavity of the tumor was filled with clotted blood.

**Fig. 4:**
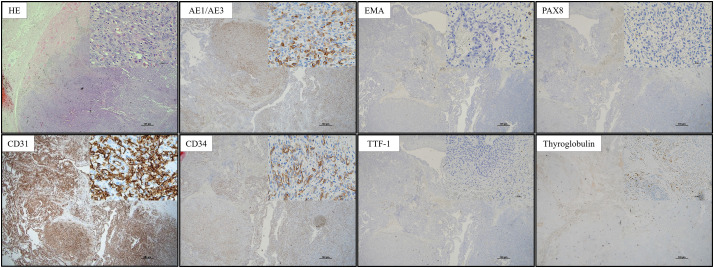
Immunohistochemical images showed that the tumor cells were positive for CD31, CD34, and AE1/AE3 and negative for EMA, PAX8, TTF-1, and thyroglobulin. EMA, epithelial membrane antigen; HE, hematoxylin-eosin; PAX8, paired box 8; TTF-1, thyroid transcription factor-1

He underwent a right thyroid lobectomy, combined resection of anterior neck muscles, and the skin surrounding the papules. The operation time was 214 minutes, with blood loss of 276 mL. The tumor contents extruded around the post-fine-needle aspiration papules in the anterior neck, but no tumor invasion was observed in other surrounding tissues. The right recurrent laryngeal nerve was intact. The right superior parathyroid gland was preserved, and the right inferior parathyroid gland was transplanted into the left sternocleidomastoid muscle. The postoperative course was good, and the patient was discharged home on the 3rd POD. The resected specimen revealed a highly hemorrhagic, brown tumor protruding from the right thyroid lobe (**Fig. 3**). Histologically, HE staining atypical cells with enlarged nuclei, prominent nucleoli, and increased chromatin formed papillary, solid, and partially erythrocyte-containing tubular structures. The tumor infiltrated the anterior neck muscles, surrounding adipose tissue, and the dermis of the resected skin, but there was no clear exposure of the tumor margin. The resected specimen has no normal thyroid component. Immunohistochemically, the tumor cells were diffusely positive for CD31, focally positive for CD34, positive for AE1/AE3, negative for EMA, PAX-8, TTF-1, and thyroglobulin, consistent with angiosarcoma (**Fig. 4**).

**Fig. 5:**
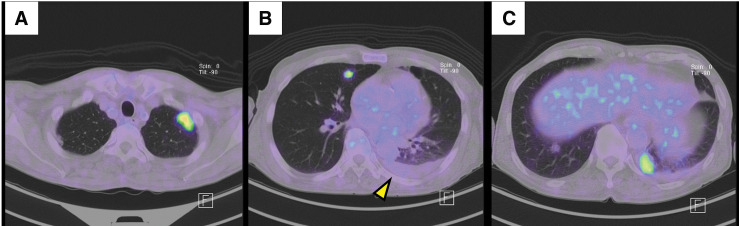
(**A–C**) Multiple pulmonary nodules uptook FDG, and left pleural effusion increased (yellow arrowhead). FDG, ^18^F-fluorodeoxyglucose

**Fig. 6:**
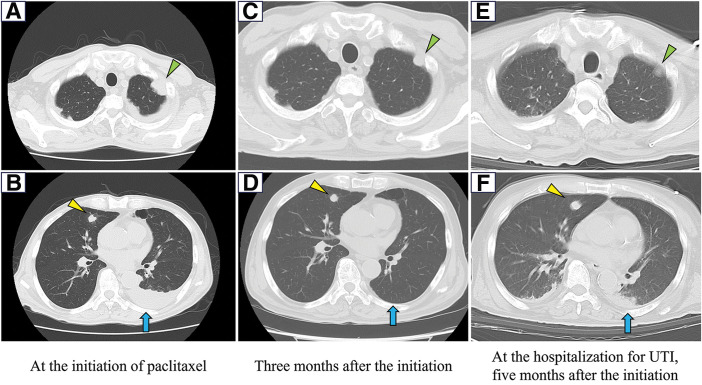
(**A**, **B**) A chest CT image taken on the 1st day of paclitaxel administration showed metastatic nodules in the left upper lobe (green arrowhead) and right upper lobe (yellow arrowhead), and pleural effusion (blue arrow). (**C**, **D**) A chest CT image taken at 3 months after the initiation of paclitaxel showed a shrinking nodule in the left upper lobe (green arrowhead) and a nodule with no change in the right upper lobe (yellow arrowhead), and decreased pleural effusion (blue arrow). (**E**, **F**) A chest CT image taken at the hospitalization for UTI, 5 months after the initiation of paclitaxel, showed the nodules with no change in the left upper lobe (green arrowhead) and right upper lobe (yellow arrowhead), and a small amount of pleural effusion (blue arrow). UTI, urinary tract infection

An FDG-PET scan was performed to determine whether the tumor was primary or metastatic. The scan revealed multiple new pulmonary nodules uptaking FDG, but there were no other areas of increased FDG uptake. Left pleural effusion increased (**Fig. 5**). Therefore, we diagnosed this disease as TAS with pulmonary metastasis. Since there is no standard treatment for TAS, we conducted FoundationOne^®^ CDx testing, which yielded no therapeutic recommendations (**Table 2**). Two months after the surgery, weekly paclitaxel therapy (100 mg/m^2^) was initiated. The pulmonary nodules and pleural effusion decreased and remained stable during the therapy of paclitaxel (**Fig. 6**). Five months after the initiation of paclitaxel, the patient was hospitalized for a UTI and treated with antibiotics. The patient never developed neutropenia during paclitaxel therapy. CT scans at the hospitalization showed no enlargement of pulmonary nodules, and no new metastatic lesions. The CT scan showed a small bilateral pleural effusion, but we considered that it was transudative pleural effusion due to UTI (**Fig. 6**). We determined the disease to be Partial Response based on Response Evaluation Criteria in Solid Tumours (RECIST) 1.1. Although blood tests on admission showed no neutropenia, the UTI worsened, and the patient died from septic shock.

**Table 2:** Result of FoundationOne^®^ CDx

Microsatellite status	Stable
*Tumor Mutation Burden*	0 Mutations/Megabase
*BCORL1*	A1068fs*12
*CD274 (PD-L1)*	Amplification-equivocal
*JAK2*	Amplification-equivocal
*KDM6A*	R519*-subclonal
*PDCD1LG2 (PD-L2)*	Amplification-equivocal
*PTEN*	L140F

## DISCUSSION

Primary TAS is a rare tumor entity that was recognized in the World Health Organization classification in 2013.^8)^ Clinically, TAS typically presents as a painless, infiltrative mass causing cervical swelling. In severe cases, rapid tumor growth can lead to compressive symptoms such as dyspnea, hoarseness, and dysphagia.^9)^ TAS has been reported to be associated with iodine deficiency and multinodular goiter. However, this case occurred in Japan, an iodine-replete region, and the tumor was a solitary nodule.^10)^

Histologically, TAS exhibits extensive central necrosis and hemorrhage, with tumor cells located at the periphery of the lesion. It is often difficult to differentiate between anaplastic thyroid carcinoma (ATC) and angiosarcoma of the thyroid, both of which are highly malignant thyroid tumors. The cells of TAS express various endothelial markers, among which CD31 and erythroblast transformation-specific related gene (ERG) are useful positive markers for diagnosing angiosarcoma. CD34 and von Willebrand factor VIII-related antigen are endothelial markers that show variable expression in TAS.^11,12)^ Notably, CD31 and ERG have been reported to be particularly useful for a diagnosis of TAS.^13)^ In TAS, PAX-8, TTF-1, and thyroglobulin are often negative. On the contrary, PAX-8 positivity is very useful for the diagnosis of ATC.

Regarding genetic mutations in TAS, some reports indicate a lower frequency of TP53 and TERT promoter mutations, which are commonly observed in undifferentiated carcinoma, while the incidence of PIK3CA mutations is comparable. This suggests a distinct tumor origin for TAS compared with undifferentiated carcinoma.^13)^ In this case, we performed a comprehensive genomic profiling test, which did not detect mutations in PIK3CA, TP53, or TERT. No other gene-specific tests were performed.

The optimal treatment strategy for primary TAS remains undetermined due to its extreme rarity and the lack of prospective evidence. Although combining radical resection with adjuvant radiotherapy has shown potential for improving outcomes and survival rates, these findings should be interpreted cautiously.^14–18)^

Surgical treatment typically involves total thyroidectomy, however, the extent of tissue invasion varies, leading to a lack of consensus on the optimal surgical approach.^19)^

Radiotherapy has been exploited in some TAS patients, with case reports demonstrating favorable outcomes. Nevertheless, a standardized approach has yet to be established.^19)^

As for (neo)adjuvant therapy, the role of chemotherapy is still evolving. Single-agent or combination regimens, including ifosfamide, anthracyclines, and taxanes are frequently used (**Table 3**),^5,16,20–33)^ but individual patient backgrounds vary, and the therapeutic efficacy is limited. In addition, the standard therapy for metastatic TAS has not been decided yet. Since we had no prior experience treating thyroid angiosarcoma or other angiosarcomas, we consulted with a multidisciplinary team in our hospital. But they also had no experience with the treatment of TAS. Although we considered an anthracycline, the patient preferred a taxane due to his advanced age. The reason why weekly paclitaxel therapy was selected for this patient was that it is considered the 1st-line treatment for cutaneous angiosarcoma, and its efficacy has been recognized.^34,35)^

**Table 3:** Previous case reports about chemotherapy for TAS

Author	Year	Country	Patient (n)	Treatment	Regimen (n)	Follow-up status
Rhomberg^16)^	2004	Austria	9	Surgical resection + adjuvant CHT(1) Surgical resection + adjuvant CRT(8)	RAZ(6)	Median: 48.5 months (range, 0.5–196 months)
					RAZ + VND(2)	Mean: 13.75 months
					DOX + VND(1)	1 month
Yilmazlar^20)^	2005	Turkey	1	Total thyroidectomy + adjuvant CHT	IFO + DOX	12 weeks, DED
Fulciniti^21)^	2008	Italy	1	Total thyroidectomy + adjuvant CHT	Details unknown	15 months
Isa^22)^	2009	Malaysia	1	Hemithyroidectomy + CHT	PAC	7 months, DED
Binesh^23)^	2011	Iran	1	Neoadjuvant CHT + RT	IFO + DOX	4 months, DED
Stacchiotti^24)^	2012	Italy	1	Neoadjuvant CHT	GEM	14 months
Innaro^25)^	2013	Italy	1	Total thyroidectomy + adjuvant CHT	IFO + DOX+ dacarbazine + mesna	22 months, NED
Collini^5)^	2016	Italy	5	Surgical resection + neoadjuvant CHT(1)	PAC + IFO + EPI	36 months, DED
				Surgical resection + adjuvant CHT(3)	IFO + DOX as adjuvant and PAC after 2nd surgery at relapse	9 months, DED
				Surgical resection + adjuvant CRT(1)	IFO + EPI	82 months, NED
					PAC for 1 cycle and GEM for 3cycles as neoadjuvant, and IFO + EPI as adjuvant	70 months, NED
					IFO + EPI as adjuvant after 1st surgery and neoadjuvant before 2nd surgery at relapse	59 months, NED
Crawford^26)^	2017	USA	1	Total thyroidectomy + adjuvant CHT	PAC	14 months, NED
Marina^27)^	2018	Italy	1	Total thyroidectomy + adjuvant CHT	IFO + EPI + mesna	62 months, NED
Kondapalli^28)^	2019	USA	1	Total thyroidectomy + adjuvant CRT	IFO + DOX + mesna	23 months, NED
Bravaccini^29)^	2021	Italy	1	Hemithyroidectomy + CHT	DOX	6 months
Probst^30)^	2021	Germany	1	Total thyroidectomy + adjuvant CHT	PAC	4 months, DED
Bala^31)^	2022	Portugal	1	Total thyroidectomy + adjuvant CRT	PAC, DOX, GEM, docetaxel, and pazopanib	29 months, DED
Lu^32)^	2023	China	1	Subtotal thyroidectomy + adjuvant CHT	IFO + EPI	31 months, NED
Gurluler^33)^	2024	Turkey	1	Hemithyroidectomy + CHT	PAC	Unknown, DED
Our case			1	Hemithyroidectomy + CHT	PAC	5 months, DED

CHT, chemotherapy; CRT, chemoradiotherapy; DED, died of disease; DOX, doxorubicin; EPI, epirubicin; GEM, gemcitabine; IFO, ifosfamide; NED, no evidence of disease; PAC, paclitaxel; RAZ, razoxane; RT, radiotherapy; VND, vindesine

In this case, chemotherapy was introduced immediately after confirmation of enlargement of metastatic nodules and additional increase of pleural effusion by CT. Even in the absence of clear metastasis, early initiation of postoperative chemotherapy should be considered for TAS, given its highly malignant nature.

This patient passed away from septic shock of UTI, however, no neutropenia developed during paclitaxel treatment, so it is unlikely that paclitaxel caused the patient’s death.

Currently, new therapeutic strategies target vascular endothelial growth factor, vascular endothelial growth factor receptor, and PI3K pathway, which are considered potential targets for optimizing treatment for TAS.^36–39)^ However, effective results have yet to be achieved.

Further research on multidisciplinary treatment strategies for TAS is required.

## CONCLUSIONS

We have reported on the perioperative management of TAS. In the absence of established standard treatments, tumor resection combined with paclitaxel may potentially inhibit disease progression. We suggest that large or enlarging thyroid tumors should be surgically resected. Although this is a single-case report, we hope it will contribute to the diagnosis and standard treatment of TAS. Further case reports of TAS are needed to establish optimal treatment strategies.
